# Peanut Butter Food Safety Concerns—Prevalence, Mitigation and Control of *Salmonella* spp., and Aflatoxins in Peanut Butter

**DOI:** 10.3390/foods11131874

**Published:** 2022-06-24

**Authors:** Tapiwa Reward Sithole, Yu-Xiang Ma, Zhao Qin, Xue-De Wang, Hua-Min Liu

**Affiliations:** College of Food Science and Engineering, Henan University of Technology, Zhengzhou 450001, China; tappsithole@gmail.com (T.R.S.); myx366@haut.edu.cn (Y.-X.M.); qinzhao@haut.edu.cn (Z.Q.); hmliu@haut.edu.cn (H.-M.L.)

**Keywords:** peanut butter, peanut food safety, *Salmonella* spp., aflatoxins

## Abstract

Peanut butter has a very large and continuously increasing global market. The food safety risks associated with its consumption are also likely to have impacts on a correspondingly large global population. In terms of prevalence and potential magnitude of impact, contamination by *Salmonella* spp., and aflatoxins, are the major food safety risks associated with peanut butter consumption. The inherent nature of the *Salmonella* spp., coupled with the unique chemical composition and structure of peanut butter, present serious technical challenges when inactivating *Salmonella* spp. in contaminated peanut butter. Thermal treatment, microwave, radiofrequency, irradiation, and high-pressure processing all are of limited efficacy in inactivating *Salmonella* spp. in contaminated peanut butter. The removal of aflatoxins in contaminated peanut butter is equally problematic and for all practical purposes almost impossible at the moment. Adopting good manufacturing hygiene practices from farm to table and avoiding the processing of contaminated peanuts are probably some of the few practically viable strategies for minimising these peanut butter food safety risks. The purpose of this review is to highlight the nature of food safety risks associated with peanut butter and to discuss the effectiveness of the initiatives that are aimed at minimising these risks.

## 1. Introduction

### 1.1. Status of the Peanut Butter Market 

In recent years the peanut butter market has been on a steady increase and is further projected to continue on this positive trajectory. The compounded annual growth rate (CAGR) of the global peanut butter market increased by 6.1% between 2017 and 2021 [1]. As of 2021, the global market value of peanut butter was USD 3.4 Billion and was projected to undergo a moderate growth of between 4.4–10% by 2027 [2,3,4]. Recently, the large entities in the peanut butter industry significantly expanded their operations and product offerings [2,5,6]. 

The USA is the world’s largest peanut butter consumer by volume where is it found in 90% of the households. The peanut butter market even experienced further gains during the COVID-19 pandemic [7,8]. In the US alone, in 2020, about 87.9 million people were recorded to have consumed at least one jar of peanut butter (462 g) within a space of 1 month [9]. In Europe, from 2013 onward the total peanut butter and prepared/preserved groundnuts output volume increased consistently by about 3.2% every year reaching its peak in 2019, thereafter decreased by about 1.5% in 2020 due to the effects of COVID-19 [10,11]). This was attributed to the fact that the EU peanut butter markets strongly depend on peanuts and peanut butter imports [10,11]. However, it is projected to return to pre-COVID-19 pandemic levels where peanut butter imports to the EU had almost doubled from 26,000 metric tons to 40,000 metric tonnes between 2014 and 2018 [10]. Further, it is interesting to note that in the UK, whereas jam spread has been very popular in the past, the sales of peanut butter spreads exceeded that of jam spread in 2020 [7].

### 1.2. The Food Safety Risk Associated with Peanut Butter 

Associated with peanut butter, microbial contaminations specifically by *Salmonella* spp., and biological toxins in the form of aflatoxin are the main peanut butter food safety concerns that have consistently been reported over the past years [12,13,14,15]. While these critical and generic peanut butter food safety concerns (*Salmonella* spp., aflatoxins) are equally problematic for both developed and developing counties, the actual scope of concern differ widely with the level of development of a country [16,17]. In most developed countries, peanut butter production is dominated by large food industry corporates [8,18]. With bigger corporates, enforcement and adherence to food safety standards is usually achievable [17]. Most of the big food corporates have adopted at least one if not all of the following: Grocery Manufacturers Association (GMA) guidelines, Good Agricultural Practices (GAPs), Hazard Analysis of Critical Control Points (HACCP) or International Organization for Standardization (ISO) as a benchmark for food quality assurance [16,18,19]. With the correct and consistent adherence to these food standards, coupled with a strong food regulations legislative enforcement, the chances of occurrence of food safety incidences are considerably lowered in the developed countries. In this regard, the general peanut butter food safety concern is not about heightened chances of occurrence but rather that in the event of contamination occurring at such a large production facility the magnitude of the potential effects on the population is very large and can result in some serious economic ramifications. While in developing counties where the market is dominated by small scale producers some of them unregulated, the scope of concern shifts from magnitude of risk to increased likelihood and frequency of occurrence due to poor monitoring and poor hygienic practises [12,17,20,21]. Apparently, both scenarios are likely to conceal, from the public, the actual significance of the food safety risk posed by peanut butter. In developed countries, people are likely to downplay the significance of these risks despite the magnitude of scale of pervious outbreaks, most likely because the occurrences have been remote and spaced out in time. For example, in 2009 in the US after a CDC warning and a major peanut butter recall, peanut butter saw a dip in sales and use, but returned to previous year levels just within 4 months [22]. Again, after the 2007 Peter Pan peanut butter recall, Bakhtavoryan, Capps, & Salin [23] reported that apart from the increased competition, there were no statistically significant differences in price elasticities for the affected brand, and just in 27 weeks, the brand had essentially recovered from the food safety crisis. For developing counties, despite the high frequencies of sporadic occurrences, peanut butter safety aspects escape public attention because the magnitude is usually small and geographically distributed coupled with poor monitoring, poor reporting systems, general limited knowledge and awareness of the consumers, and general lack of resources [20,21,24,25,26]. Notwithstanding this, cumulatively for both scenarios, the peanut butter food safety risk might be quite substantial. 

It is clear that due to the large market size and the increasing popularity of peanut butter, the likelihood of subsequent food safety risks associated with peanut butter can be of great interest to a significantly large percentage of the general global populace and to the food safety authorities by extension. Taking into account as well the market size of the other peanut butter based products, any food safety issues associated with peanut butter are likely to have much larger spill-over effects on the global food market. Apart from the potential magnitude of risk, equally worrisome is the fact that once contamination occurs in peanut butter, decontaminating it becomes extremely difficult. Furthermore, the fact that peanut butter is sold as a ready-to-eat food product requires special attention to aspects of its food safety. This paper elucidates the main food safety concerns associated with peanut butter that may be otherwise obscured or erroneously downplayed in the public domain. In addition, we present some specific technical details on the challenges to decontaminating contaminated peanut butter and recent developments that have been aimed at minimising the associated risks. 

## 2. The Food Safety Risks of Butter Contamination by *Salmonella* spp. 

**Figure 1:**
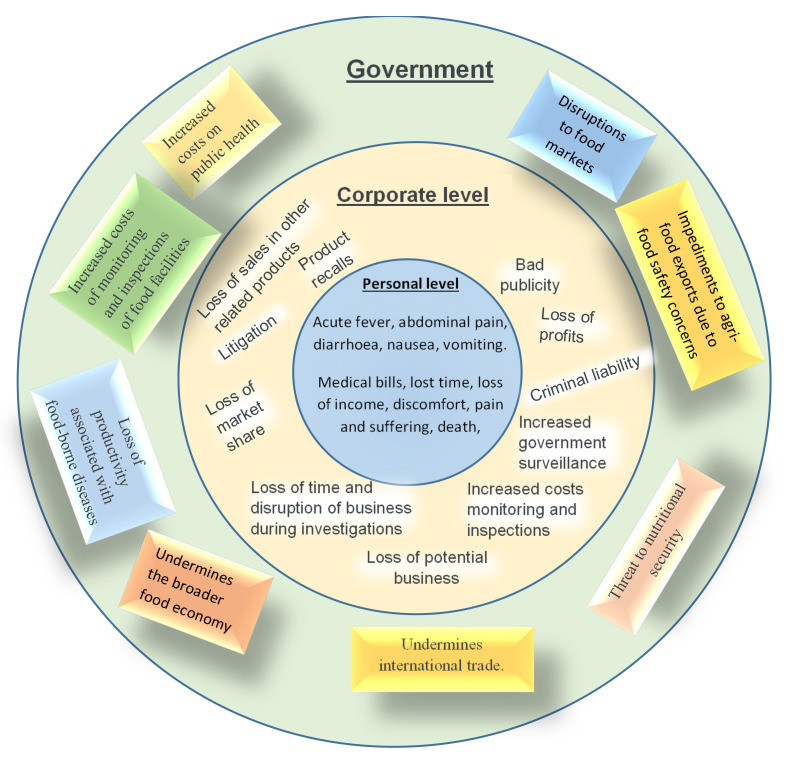
Effects of Salmonella spp. pandemics at personal, company and government level. Approximate magnitude of the monetary value of costs increase from the centre outwards.

Globally, it is estimated that each year around 93.8 million cases of gastroenteritis occur due to *Salmonella* spp. and result in approximately 155,000 deaths [27]. While egg-based and chicken dishes are the most common food vehicle associated with the *Salmonella* spp. outbreaks, *Salmonella* spp. contamination in low moisture foods, peanut butter, among others, also tops the list of the most problematic and challenging food safety risks [28,29,30,31,32,33]. In the past it was erroneously assumed that peanut butter is not a high-risk food product in terms of microbial contamination [18,19,29]. This could have been partly premised on the understanding that with a moisture content of about 6.5% in dry peanuts; which goes further down to 3% after roasting; and a typical water activity of less than 0.35 in finished peanut butter, the proliferation and growth of most spoilage and pathogenic microbes within the entire peanut butter production chain is substantively inhibited [34,35,36]. Moreover, given that optimum water activity for growth of *Salmonella* spp. is about 0.99, it might be rational to conclude that at a water activity of about 0.35, finished peanut butter is generally safe from *Salmonella* spp. [37]. Furthermore, prior to grinding, peanuts are typically first roasted at 140 °C for about 20 min. This process has the potential to inactivate almost most of the microbes [38]. Thus, in most cases, peanut butter contamination is due to contamination or re-contamination of peanut butter in processes that come after roasting. Notwithstanding, some *Salmonella* spp. outbreaks directly linked to peanut butter have been recorded. In 1998 a *Salmonella* Mbandaka outbreak was reported in South Australia. In 2007, a *Salmonella* Tennessee outbreak in the USA resulted in 715 reported cases across 48 states. In 2009, 714 cases of *Salmonella* Typhimurium in the USA were reported across 46 states with 9 deaths and in 2012, *Salmonella* Brendeney was reported to have infected 42 people across 20 states. In 2014, a *Salmonella* Braenderup outbreak affected 6 people in 5 states [39,40,41,42,43]. When such *Salmonella* spp. outbreaks linked to peanut butter occur, they have far-reaching effects, cutting across individual to national or even international level at times. In the US for example, *Salmonella* spp. outbreaks linked to peanut butter resulted in reported hospitalisations and deaths, massive peanut butter recalls, recalls of over 3900 other products that use peanut butter as an ingredient, bankruptcy of the Peanut Corporation of America (PCA), criminal prosecutions, conviction and imprisonment sentencing of PCA top management, 8 million criminal fine and 3.2 million forfeiture of assets of ConAgra Foods Inc., and enactment of a new law by the US federal government (Food Safety Modernisation Act) [22,44,45,46]. A summary of possible wide-ranging impacts that can result from *Salmonella* spp. outbreaks in the peanut butter industry is shown in Figure 1.

It is no doubt therefore that *Salmonella* spp. outbreaks in peanut butter pose a serious food safety threat, significant economic impacts and substantial social consequences [47]. 

### 2.1. Technical Challenges of Inactivating Salmonella spp. in Peanut Butter 

Over and above the possible impacts on public health and the economy, peanut butter contamination poses unique and serious technical challenges with respect to the inactivation of *Salmonella* spp. in the contaminated products [28]. This challenge emanates from the combined effect of the inherent nature of *Salmonella* spp. as a bacteria and the unique structure and chemical composition of peanut butter. Several *Salmonella* spp. inactivation methods that are ordinarily applicable and effective for other bottled products fail when it comes to peanut butter mainly on grounds of low efficacy and the potential of generating undesirable changes to flavour. Further, it has been reported that once contamination occurs, *Salmonella* spp. is able to remain viable even throughout the entire shelf life of the peanut butter [34,48,49] 

### 2.2. Nature of Salmonella spp. 

*Salmonella* spp. by nature is a hardy, ubiquitous, facultative anaerobic, non-spore-forming gram-negative bacilli, rods genus bacterial, capable of surviving for several months in water and even for years in dry environments [50,51]. Under favourable conditions of temperature, humidity, and pH, *Salmonella* spp. exhibits great versatility, adapting to different hosts and mediums such as soil and water [33,52]. It is also reported to be capable of undergoing a cyclic lifestyle characterised by passage through a host into the environment and back into a new host after several years [53]. In one such case, the US Food and Drug Administration (US FDA) [54] reported a *Salmonella* Agona outbreak that was caused by the use of contaminated water in a dry cereal plant in Midwest United States in 1998. The use of the contaminated water in cereals in that plant was corrected and the water used to mix mortar for construction renovations in the plant. A total of 10 years later, in 2008, new renovations disturbed the 1998 brick and mortar of the previous renovations and another *Salmonella* Agona outbreak immediately resurfaced again in that plant. Whole-genome sequencing (WGS) of *Salmonella* spp. isolates samples obtained from the cereals, and the environmental samples obtained from that plant confirmed that exactly the same *Salmonella* Agona strain that was found in water 10 years ago had now resurfaced after such a long period of dormancy in the plant walls. Such is the resilience of *Salmonella* spp. even in such unlikely environments. 

In food ingredients, pre-exposure of *Salmonella* spp. to abiotic stressors increases the chances of developing cross-tolerances to several potential future stressors leading to even more virulent cells in the final food product [33]. Pre-exposure to abiotic stressors is common when peanuts are still in the shell during pre-harvest environments, in curing steps, and during roasting [35]. Such pre-exposures subsequently confreres to *Salmonella* spp. even an increased resilience and survivability in low moisture and high-fat foods such as peanut butter [35,47,55]

In general, a combination of low moisture and high sugar and or fat content in a food product such as peanut butter and other low moisture foods is believed to contribute to an enhanced survival and heat resistance of *Salmonella* spp. in such foods [28]. *Salmonella* spp. can survive in low-moisture (<0.83 a_w_), high-protein, and high-fat foods for several years; furthermore, it is capable of surviving for longer periods at low-temperature storage [36]. For example, when *Salmonella* spp. was inoculated in tahini (sesame butter product), and stored for 119 days at 4 °C, it remained viable and did not show any substantial reduction in population over the entire storage period [56]. Burnett, et al. [48] further assert that depending on the formulation, post-process contamination of peanut butter and spreads is even more problematic in that *Salmonella* spp. might survive in these products for the entire duration of their shelf life at 5 °C and also possibly at 21 °C as well. More so, once a bacterium such as *Salmonella* spp. has contaminated a dry-food production environment such as a peanut butter production facility, its subsequent removal from the plant can also prove to be very challenging [28]. 

### 2.3. Adaptation of Salmonella spp. in Stress Environments 

The mechanism of adaptation and survival of *Salmonella* spp. under conditions of low water activity such as in peanut butter is not yet completely understood. It is suggested that the survival of *Salmonella* spp. during desiccation in the absence of nutrients and at low osmolality is enhanced by its ability to express specific red, dry and rough (rdar) morphotype, characterised by multicellular patterned aggregative colonies. [53,57,58]. The basic structural components of the rdar morphotype are the curli fimbriae and cellulose. Curli fimbriae facilitates surface adhesion and intercellular aggregation of bacteria, whilst the cellulose (and other exopolysaccharides) promotes intercellular interactions [53,58]. He, et al. [35] suggests that some morphological alterations (decrease in cell diameter) as shown in *Salmonella* Enteritidis, *Salmonella* Typhimurium, and *Salmonella Tennessee* under desiccation stress in low water activity peanut oil could possibly be linked to their stress adaptation and survival mechanism. These morphological changes might be the contributory factor to the increased resilience of desiccation-stressed *Salmonella* spp. in other environmental challenges such as heat [35].

Lee, Shoda, Kawai, & Koseki [59] proposed that one of the factors that enhance long-term survival and environmental stress tolerance of *Salmonella* spp. in low water activity environment is vitrification of bacterial cells by the glass transition phenomenon. When *Salmonella* spp. bacterial vitrify due to a decrease in temperature and low water activity, there is limited molecular movement and physical properties similar to a solid are exhibited. This change in state characterised by low molecular movement is likely to confer to *Salmonella* spp. increased tolerance to various environmental stresses such as heat, desiccation, pressure, and water activity [59]. Using thermal rheological analysis (TRA) Lee, et al. [59] managed to determine softening behaviour associated with this state change and thus determined the glass transitional temperatures (*T_gs_*) of some five strains of *Salmonella* spp. (*S*. Typhimurium, *S*. Chester, *S*. Oranienburg, *S*. Stanley *S*. Enteritidis). It was observed that *T_gs_* of these 5 strains analysed a range between 35.16 °C to 57.46 °C at 0.87 a_w_, and 77.10 °C to 83.30 °C at 0.43 a_w_ demonstrating that *T_g_*_s_ increased as the a_w_ decreased for all the 5 tested *Salmonella* spp. *enterica* serovars. Abdelhamid & Yousef [60] concluded that under conditions of low moisture, *Salmonella* spp. enters a viable but nonculturable (VBNC) state and that this might be the possible avenue of its survival in low water activity foods. In the VBNC state, the bacteria will be metabolically dormant and thus resilient to stressors. Further, they demonstrated that when the 2 *Salmonella* spp. enterica serovars that are known to acquire desiccation resistance (Tennessee and Eimsbuettel) were subjected to the same dehydration stress for 72 h, serovar Tennessee increased the biofilm-forming ability but Eimsbuettel did not. Furthermore, in their study, a 2-day storage of desiccation-adapted cells at 4 °C resulted in significantly high upregulating (>2-fold increase) of 4 desiccation-related genes, proV, STM1494, kdpA, and otsB and (>50-fold increase) of the universal stress response regulator, rpoS. Extended storage at the same temperature storage for 14 days increased the expression of proV and rpoS genes while 2 virulence regulatory genes, hilA and invA, was downregulated (>2-fold decrease) at both storage periods [60].

Apart from the phenotypic adaptation mechanisms another possible adaption strategy of *Salmonella* spp. under conditions of stress could possibly be through genetic mutations [61,62]. When *Salmonella* Agona (ATCC 51,957) and *Salmonella* Mbandaka NCTC 7892 (ATCC 51,958) strains were subjected to repeated heating and drying under a high fat and low moisture matrix, genetic differences increased with every heat treatment, however, no increased fitness of the strains was observed, even after 10 repeated heat treatment cycles [63]. Fortunately or unfortunately mutational adaptation is often deleterious, and can result in decreased fitness in a population [64]. While high mutation rates result in quick adaption the resultant loss of fitness enables non-mutators to outcompete mutators in a given environment in the long run [64]. Thus, the long-term survival of bacteria under an environment of stress might be influenced more by non-mutational adaptation strategies [64]. 

### 2.4. Nature of Peanut Butter

The structure and chemical composition of peanut butter present unique challenges to the inactivation of *Salmonella* spp. in contaminated products. When compared with some other nut butters having similar matrixes and comparable water activities, *Salmonella* spp. appears to show even greater resistance to thermal inactivation in peanut butter (peanut > almond > hazelnut) [65]. More than 60 min of holding at 90 °C was required to achieve a 5 log CFU/g reduction in *Salmonella* spp. in peanut butter whereas just about 30 min sufficed to achieve the same 5 log CFU/g reduction in hazelnut and almonds nuts butter formulations [65]. The differences may emanate from the different chemical compositions of the nut matrixes thereby conferring the *Salmonella* spp. in the peanut butter some additional resistance to thermal inactivation. Peanut butter is a colloidal suspension of lipid and water within a peanut meal phase [28,48]. The actual composition of peanut butter itself varies widely with product formulation. As shown in Table 1, even the standards that are used for peanut butter also vary. 

While in the EU the use of the label “peanut butter” might not be strictly regulated by law, in the US, the Federal Regulation specifically stipulates that all commercial peanut products sold with the label “Peanut Butter” must contain at least 90% peanuts, if less than 90% peanut content, it must be labelled “peanut butter spread” [69]. A product that does not comply with the provisions of this peanut butter standard must be labelled “Imitated Peanut Butter” [69]. In general, a typical formulation of commercial peanut butter contains 90–95% of roasted, blanched and ground peanuts passing 200 mesh screens, 1.5% salt, 0.125% hydrogenated oils, 2% dextrose, 2–4% honey or corn syrup [70,71]. While the final chemical composition of peanut butter depends on the product formulation, values 45.6 to 51.1 for crude fats, 19.5 to 24.2% for proteins, 24 to 32% carbohydrates, 2.11 to 4.46% fibre, 3.16 to 3.26% ash, 0.54 to 0.74% moisture and a pH of about 6.1 to 6.4 can be taken as rough guides [48,72,73]. Apart from the differences in the chemical composition of peanut butter on the market, substantial differences in the structure of the peanut butter also exist between creamy and crunchy peanut butter. 

### 2.5. Influence of Product Formulations on the Efficacy of Inactivation of Salmonella spp. in Contaminated Peanut Butter 

When predicting the inactivation of *Salmonella* spp. in peanut butter compositional factors must be accounted for [36]. Differences in product formulation result in significantly different *Salmonella* spp. inactivation kinetics in peanut butter [19,36]. Burnett, et al. [48] compared the inactivation kinetics of *Salmonella* spp. in 5 different commercial peanut butter formulations (more than 90% peanut) and two peanut butter spreads (less than 90% peanut content). After 24 weeks of storage, it was observed that the order of retention of the viability of *Salmonella* spp. in these samples was significantly different: peanut butter spreads > traditional (regular) and reduced sugar, low-sodium peanut butter > natural peanut butter (no stabiliser added) [48]. Li, Huang, & Chen [74] reported that when three peanut butter samples; Omega 3 fortified, regular fat, reduced sugar, (all with approximately 50% fats), and 1 reduced-fat peanut butter spread (with approximately 33.3% fats) were inoculated with a cocktail of *Salmonella* spp. and heated at 70, 75, 80, 85, and 90 °C, the least thermal resistance of *Salmonella* spp. was found in the samples fortified with Omega 3, while the highest was found in the sample with reduced-fat. On the other hand, no appreciable difference in bacterial thermal resistance was observed between reduced sugar (18.9% carbohydrates) and regular fat (21.9% carbohydrates) formulations. He, et al. [34] also observed significantly different D-values for the inactivation of *Salmonella* spp. in peanut butter of different formulations; regular, organic, and reduced-fat peanut butter. Over a 4-week storage period and at the same water activity of 0.4, regular peanut butter sample with 33.33% fat and 41.67% carbohydrate resulted in less than a 1-log reduction in the total *Salmonella* spp. count, while organic peanut butter with more fat (50%) but less carbohydrate (21.88%) had a higher bacterial count log reduction [34]. The inactivation kinetics of *Salmonella* spp. at both refrigerated (4 °C) and room storage condition (25 °C) at the same water activity (0.4) appears to show that high-fat low-carbohydrates peanut butter results in higher *Salmonella* spp. inactivation in comparison to low-fat high-carbohydrates [34]. 

The combined effects of composition, water activity, and temperature results in significantly different (*p* < 0.05) thermal resistance of *Salmonella* spp. in peanut butter formulations [36]. Further, the combined influence of peanut butter composition, water activity, and temperature on resistance of *Salmonella* spp. to inactivation is even far complex to draw simple generalisations. Jin, et al. [36] demonstrated that even in foods that are made of exactly identical ingredients, differing only in relative percent compositions of those same ingredients, such that one can be characterized as high fat and the other as high protein, significant differences in *Salmonella* spp. inactivation kinetics will be observed even when both are at the same water activity. After comparing the D-values for *Salmonella* spp. in high protein against the high fat model matrix, it was established that above a certain temperature (79.48, 71.28, 69.62, and 38.428 °C) value, depending on the water activity a_w_ (0.63, 0.73, 0.81, and 0.90, respectively), the D-values of *Salmonella* spp. are higher in a high-protein matrix in comparison to a high-fat matrix, while below those temperature values the D-values for higher protein matrix will be less than those in high-fat matrix [36]. 

However, in general though, it appears that a natural peanut butter formulation (peanuts and small amounts of salt only) and higher fat formulations provide a microenvironment that is least favourable for *Salmonella* spp. survival. Burnett, et al. [48] highlight that cells of *Salmonella* spp. tend to aggregate and clump within or near the water phase in the colloidal suspension of peanut butter or peanut spreads. The differences in the rate of inactivation could possibly be due to the differences in the size of the lipid and water droplets dispersed in the meal. Nutrient availability is then a function of the cell density within or around the water phase [48]. With bigger water droplets and a higher cell density around the water phase, nutrient availability is likely to be low, thus resulting in unfavourable conditions for the continued survival of *Salmonella* spp. This provides a possible explanation as to why in natural peanut butter (without stabilisers) where a coalescence of the dispersed water droplets is more pronounced, the survival of *Salmonella* spp. with storage is less than in stabilised peanut butter. It is clear, therefore, that product formulation has a significant effect on the survival of *Salmonella* spp. in peanut butter. Thus, for the effective inactivation of *Salmonella* spp. in peanut butter or any peanut butter product, it is of paramount importance to take into cognisance the composition of the peanut butter or the peanut butter-based food product. A general inactivation treatment design short of such special considerations might not suffice.

### 2.6. Thermal Inactivation of Salmonella spp. in Contaminated Peanut Butter 

Attempts to inactivate *Salmonella* spp. in contaminated peanut butter have been undertaken by several researchers with thermal inactivation topping the list, albeit with limited success [65,75,76,77]. In most operations, the peanut roasting stage is primarily aimed at improving the organoleptic properties of peanuts, and at the same time the high temperatures also double up as the only substantial microbial kill step. However, some commercial peanut butter operations might consider an additional microbial thermal kill step just before final packaging to cater for contamination that could have possibly occurred post the roasting stage. In such cases peanut butter undergoes thermal pasteurisation at temperatures of 70–75 °C for 20 min [78]. Some studies appear to dispute the effectiveness of this procedure, especially in cases where contamination levels are high. For example, thermal treatments at 90 °C for a time period of even up to 30 min have been found to be inadequate in achieving a 5 log CFU/g *Salmonella* spp. reduction in contaminated peanut butter as is required by the US FDA regulations [19,65,75,79] 

Previously stressed *Salmonella* spp. requires a more rigorous thermal treatment in comparison to freshly cultured *Salmonella* spp. For example, He, et al. [34] reports that the inactivation of stressed *Salmonella* ssp. after 30-day storage at an isothermal temperature of 90 °C required significantly more time (*p* < 0.05), approximately 5.89 to 8.84 min, to kill 90% of stressed *Salmonella* spp. Cells, whereas it only took about 2.15 to 2.33 min to achieve the same for the fresh culture. A progressively decreasing rate of inactivation with repeated treatments was observed during thermal inactivation cycles of *Salmonella* spp. in contaminated peanut butter [74,75,79]. In spite of some few observed results to the contrary [77], the isothermal inactivation kinetics of *Salmonella* spp. in peanut butter can, to a good extent, be approximated using concave upward (*β* < 1)Weibull models, characterised by rapid death during the first minutes followed by lower death rates and tailing off of the surviving microbe population [74,75,79]. Li, et al. [74] used the Weibull survival model to describe the survival curves of *Salmonella* spp. inactivation in peanut butter and obtained an average exponent (shape factor) of between 0.38 to 0.662. Care needs to be taken on the method to be used in assessing the thermal inactivation of *Salmonella* spp. in peanut butter. Some studies indicate that the conventional methods of testing survival kinetics of *Salmonella* spp. grown in liquid culture (planktonic cell growth) do not provide the same results as *Salmonella* spp. grown in a solid matrix (sessile cell growth) [80]. Given that peanut butter is low moisture food the use of cells grown on solid media may be more accurate in assessing the survival of *Salmonella* spp. at different temperatures in a low-water-activity environment such as peanut butter [80]. While a specific validation procedure of the thermal treatment for a given peanut butter is required, process designers can possibly use these thermal inactivation kinetics as a starting point for designing future effective systems for reducing *Salmonella* spp. contamination in peanut butter [19].

### 2.7. Effects of Storage Temperature on Inactivation of Salmonella spp. in Peanut Butter

*Salmonella* spp. population in contaminated peanut butter generally decreases with storage time depending on the temperature of the storage. In comparison to low temperature storage (4 °C), a higher storage temperature (25 °C) appears to have a significant bactericidal effect [34,81]. Burnett, et al. [48] observed that a low temperature storage of peanut butter, 4–5 °C for example, is likely to provide *Salmonella* spp. more chances of survival in comparison to room temperature storage say at 21 °C. When peanut butter samples inoculated with 5·68 log10 CFU/g of *Salmonella* spp. were stored for 24 weeks at 21 °C and others at 5 °C, the *Salmonella* spp. log count reduced by 4.14–4.50 log10 CFU/g and 2.86–4.28 log10 CFU/g, respectively [48]. Kilonzo-Nthenge, et al. [49] observed a *Salmonella* spp. population decreases from 4.78 CFU/g to 3.72 CFU/g after 15 weeks of storage at 4 °C. They also observed a significantly greater decrease (*p* < 0.05) in population count of *Salmonella* spp. for peanut butter stored at 25 °C in comparison to that stored at 4 °C for the 15 weeks of storage. After a 14-day storage period, Park, et al. [81] observed 0.15 to 0.65 and 0.34 to 1.29 log CFU/g reduction in samples inoculated with 10^6^–10^7^ CFU/g and stored at 4 and 22 degrees C, respectively. These results indicate the importance of storage temperature on the survival of *Salmonella* spp. with storage. They also show that post-process contamination of peanut butter may result in the survival of pathogens for the entire product shelf life, thus posing health risks to consumers [48,49].

Ideally, when storing natural peanut butter (without stabilisers), one would generally prefer to store peanut butter at refrigeration temperatures of about 4 °C to minimise the effect of oil separation and subsequent lipid oxidation. However, in the unfortunate event that *Salmonella* spp. contamination in peanut butter has taken place and the consumer is not aware, then storing the peanut butter at refrigeration conditions might be problematic as *Salmonella* spp. has a higher survival rate at 4 °C in comparison to storage at room temperatures [48,49]. Thus, to a knowledgeable consumer, this might present a dilemma of choosing between maintaining quality or being proactive with respect to food safety.

While storage at 25 °C results in more death of *Salmonella* spp. with storage in comparison to low-temperature storage, it was observed that thermal inactivation of the remaining population of *Salmonella* spp. bacteria that were initially stored at 25 °C is more difficult than inactivating the remaining population of *Salmonella* spp. in peanut butter initially stored at 4 °C. For example, when *Salmonella* spp. cells were stored for 30 days at 25 °C, more than a 2.9-log reduction in the bacterial count was observed compared to the reduction at 4 °C [34]. However, after a subsequent 1-h heat treatment at 90 °C, the reduction in the microbial count for the sample that was initially stored at 25 °C was 3.5-log lesser than what was achieved in a sample that was initially stored at 4 °C [34]. Thus He, et al. [34] concludes that even though more bacteria die when a storage temperature of 25 °C is used, the smaller number of survivors become even more hardened to any subsequent heat stress. It is not very clear, however, if the differences in extremity between the starting and final holding temperatures, in this case from 4 °C to 90 °C compared to from 25 °C to 90 °C did not also contribute to the above observed results or that there could also be another explanation. 

### 2.8. Effects of Water Activity on Thermal Inactivation of Salmonella spp. in Contaminated Peanut Butter 

Effective thermal inactivation of *Salmonella* spp. can be achieved better at higher water activity and higher temperatures [36,82]. At higher water activities (higher than 0.9), the resistance of *Salmonella* spp. to thermal treatment in peanut butter is substantially reduced [34,35]. In general, for a given peanut butter sample, a negative correlation exists between the water activity and the temperature required to obtain the same D -value [36]. For example, to achieve the same D-value in a peanut butter sample contaminated with *Salmonella* Agona, thermal treatment in an isothermal water bath at 38.42 °C was required when the peanut butter water activity was about 0.9, while a temperature of about 79.48 °C was required when the water activity was about 0.63 [36]. This shows that the water activity of the food matrix is a key factor in determining the survival of *Salmonella* spp. under conditions of thermal stress. However, while Garces-Vega, Ryser, & Marks [83] did not manage to conclusively validate their idea using their own experimental results, they argue that the water content and not necessarily the water activity should be able to give more reliable thermal inactivation kinetics in low moisture foods. Their argument is based on the premises that water activity is temperature dependent as characterized by the hysteresis between sorption states, while moisture content is not. Thus, moisture content could be a more reliable parameter when accounting for the water effects in a matrix [83]. Xie, et al. [55] demonstrated that the actual water content of the bacterial cells themselves plays a critical role in determining the thermal tolerance of the bacterial cell. Using viable freeze-dried *Salmonella* Enteritidis PT 30 with moisture content ranges of *X_W_* (7.7, 9.2, 12.4, and 15.7 g water/100 g dry solids) which were subsequently subjected to thermal inactivation at 80 °C, they observed a negative exponential relationship between the D-value and the moisture content *X_W_* of the bacterial cells [55]. These results could also possibly suggest that the ability to quickly adjust to the microenvironment, most likely through losing water or taking in some solutes, is a key factor in the survival of *Salmonella* spp. in low moisture foods when subjected to thermal inactivation. It is therefore important that when modelling survival or inactivation of *Salmonella* spp. in multicomponent foods that involving peanut butter, measurements of water activity, moisture content, redox potential, antimicrobial concentration or pH must be, by all means, reflective of the actual values of the microenvironment or the interface where the *Salmonella* spp. microbe is likely to reside [84]. 

If peanut butter is to be mixed with some other ingredients for example hot deserts, starter soups and corn porridge and there is homogeneous hydration of the peanut butter then the resistance of *Salmonella* spp. to heat treatment will be substantially reduced [85]. In a baking experiment involving peanut butter (PB)–filled pretzels and whole wheat (WW) pita chips at 0.95 water activity that were inoculated with *Salmonella* spp. and Listeria monocytogenes, it was observed that both pathogens were below the detection limit (<1 log CFU/g) after a baking process carried out at 177 °C for 25 and 30 min, respectively [86]. However, even in a peanut butter-based food mixture with overall high-water activity and undergoing high-temperature treatment, special attention is needed to ensure that the peanut butter is homogeneously distributed in that food mixture and sufficiently hydrated. Local partial variation in composition which results in crumbs of peanut butter forming, for example, crumbs of peanut butter in porridge or soup, might produce localised regions of low water activity which might confer some additional heat resistance to *Salmonella* spp. despite the overall high-water activity of the entire dish [85]. It is important, therefore, that when preparing food mixtures containing peanut butter, sufficient care must be taken to ensure a good homogeneity of the mixture and where possible employ high temperature and extended cooking times.

### 2.9. Variations in Thermal Inactivation of Various Salmonella spp. Serotypes in Peanut Butter 

Among many other *Salmonella* spp. serotypes associated with non-typhoid, foodborne gastroenteritis, *Salmonella* Typhimurium and *Salmonella* Enteridis are the most prevalent [87]. It has been observed that at moderate heat treatment within the temperatures range of 90 °C and lower water activity of about 0.20, *Salmonella* spp. serotypes show some considerable variability in their individual resistance to heat treatment [35,75]. For example, to achieve a 5-log reduction in *Salmonella* spp. count in one peanut butter formulation, 108.08 min were required for *Salmonella* Tennessee, 48.14 min for *Salmonella* Typhimurium and 66.69 min for *Salmonella* Enteritidis [35]. However, at higher temperatures of approximately 120 °C and higher, water activity the difference was apparently not that significant [35]. Ma, et al. [75] compared the thermal inactivation kinetics of three *Salmonella* Tennessee serotype that had been implicated in a previous outbreak with *Salmonella* spp. strains of other serotypes (Enteritidis, Typhimurium, and Heidelberg) (SSOS) and a clinical isolate of *Salmonella Tennessee* from other sporadic cases (STSC). It was observed that 120, 86 and 55 min at a temperature of 90 °C was required to achieve a 7-log reduction in peanut butter contaminated with the outbreak Tennessee strains, SSOS and STSC strains, respectively [75]. Approximately 120 min was needed to reduce the outbreak strains of Salmonella Tennessee by 7 log, whereas 86 and 55 min were needed for SSOS and STSC, respectively [75]. The calculated minimum times needed to obtain a 7-log reduction at 90 degrees C for the composited 3 outbreak-associated strains were significantly greater (*p* < 0.05) than those of SSOS and STSC [75]. This shows that at moderate thermal treatment, the results from thermal inactivation of one *Salmonella* spp. serotypes might not be applicable when dealing with a different serotype. 

### 2.10. Microwave and Radiofrequency Heating Inactivation of Salmonella spp. in Contaminated Peanut Butter

Microwave (MW) and radio frequency (RF) heating both involve converting electrical energy to electromagnetic radiation which then subsequently generates heat within a product [88]. The use of microwave radiation to inactivate *Salmonella* spp. in peanut butter is very promising. Compared to conventional heating, microwave radiation is likely to result in less changes in flavour and nutritional qualities. This is because of its high heating efficiency and comparatively shorter treatment times, occasioned by the fact that microwave heating does not require an intermediate medium to transfer heat and can directly penetrate the material, thus allowing for volumetric heating [37]. A 6 kW and 915 MHz microwave treatment reduced *Salmonella* Senftenberg, *Salmonella* Typhimurium and *Salmonella* Tennessee by about 3.24–4.26 log CFU/g when applied for 5 min, and no appreciable effect on acid, peroxide, or colour values of peanut butter was observed [37]. Radio frequency heating is also very promising in inactivation of *Salmonella* spp. in contaminated peanut butter. Ha, et al. [78] investigated the effects of radio frequency heating in inactivation of *Salmonella* spp. in peanut butter cracker sandwiches. Approximately 90 s of radio frequency heating at 27.12 MHz reduced *Salmonella* Typhimurium by 4.39 log CFU/g in creamy peanut butter and by 4.55 log CFU/g in chunky peanut butter without any appreciable change in colour of the peanut butter [78]. Radio-frequency (RF) heating involves the use of electromagnetic energy at frequencies between 1 and 300 MHz to generate heat in a dielectric material [78]. In response to an externally applied AC electric field, rapid heating within the product is initiated as a result of the frictional interactions of polar dielectric molecules rotating and the space charge displacement [78,88]. Such heating is usually fast and uniform as it does not depend on the rate at which heat is transferred from an external heating surface by conduction, convection and radiation as what happens in conventional heating. In comparison to MW heating, RF uses lower frequencies which then allows for much deeper product penetration and better control of the temperature in the heated product [89]. Further research might still be required to validate and optimise these processes for commercial adoption in peanut butter industries. In such optimisation processes, the dielectric properties of the peanut butter are a critical parameter for both RF and MW heating. The dielectric properties in turn depend on composition, structure, density, moisture content, the temperature of the peanut butter, and frequency of the applied alternating field [88]. Furthermore, issues of non-uniform heating on the food and container interface, whether RF heating has a sub-lethally injured effect on microorganisms, or whether RF heating can be combined with other methods to reduce RF energy input and better preserve the quality of peanut butter might still need to be settled [90].

### 2.11. Irradiation Inactivation of Salmonella spp. in Contaminated Peanut Butter 

Cobalt 60 gamma irradiation and electron beam (e-beam) irradiation in dosage range of 0 to 3 kilograys (KGy) can possibly be used to inactivate *Salmonella* spp. in peanut butter without causing appreciable changes to the flavour and texture [91,92]. Ionizing radiation works by causing ejection of electrons from atoms resulting in generation of free radicals [93,94]. When free radicals interact with microbes, they elicit structural damages in the microorganisms (membrane breakdown, DNA conformational changes, protein aggregation, etc.) which lead to physiological changes (leakages from membranes, loss of key enzymes, etc.) and result in a substantial inhibition of microbial growth and replication [93,94,95]. Effectiveness of irradiation can be influenced by water activity and temperature of the peanut butter or peanut butter product. At lower water activities such as in peanut butter, the radiolysis of water is substantially reduced, thereby decreasing antimicrobial action [92]. Ban & Kang [92] also observed that irradiation of *Salmonella* spp. contaminated peanut butter at 25 °C appears to be more effective in comparison to irradiation at 4 °C. Heavily contaminated peanut butter might require a higher dosage of irradiation; however, some problems with oxidation might then arise. At higher dosage greater than 3 KGy, e-beam irradiation produces significant dose-dependent changes in colour, texture, lipid oxidation, and protein degradation when applied to peanut butter [94]. In foods matrixes with high lipids content, depending on the composition, irradiation can result in oxidation, polymerisation, decarboxylation and dehydration of the fatty acids and lipids and the releases several other compounds [96]. The free radicals that are produced at high dosage irradiation inadvertently result is generation of highly objectionable off-flavours mostly in the form of peroxides, carbonyl compounds, and alcohols [97]. The high lipid content of peanut butter makes it highly susceptible to oxidation. When peanut butter loses flavour due to oxidation it is highly unlikely that consumers will take it lightly. A proposed method for reducing the generation of off-flavour during radiation of high lipid foods involves vacuum packaging and carrying out the radiation at subfreezing temperatures [96,98]. Howbeit, such a procedure will require even higher irradiation dosage to achieve the same *Salmonella* spp. reduction as that which is achievable at ambient temperatures [98]. WHO, FAO and other food regulatory authorities generally accept regulated and controlled use of correctly labelled, selected few, irradiated food products [96,99]. If the process were permitted for peanut butter, one huddle to the successful marketing of irradiated peanut butter is likely to be a low acceptance by consumers [100]. One of the top deciding factors for consumers’ acceptance of food products and food technologies is its perceived naturalness. Consumers, however, are not convinced about the naturalness and the safety of food irradiation [100,101,102].

### 2.12. High Hydrostatic Pressure Inactivation of Salmonella spp. in Contaminated Peanut Butter

High hydrostatic pressure processing (HHPP) has been attempted as an alternative to thermal processing for the inactivation of *Salmonella* spp. contaminated peanut butter, howbeit, also with little success [76,103]. Interests in HHPP is mainly premised on the understanding that in comparison to thermal processing, it results in minimally processed food products with better retention of the natural chemical and physicochemical properties, preservation of the natural aroma, a fresh taste and generally improved product shelf life [104,105,106]. This is important in foods such as peanut butter where excessive heat treatment can easily result in the oxidation of the lipid component and a compromise on the flavour and overall quality. The mechanisms of inactivation of microorganism by HHPP are fairly complex [107]. HPP only affects noncovalent bonds (ionic, hydrophobic and hydrogen bonds), thus in most cases the primary protein structures remain intact while some changes may occur in secondary, tertiary and quaternary structures [108]. Usually, it is the cell membrane that is affected by high pressure processing, resulting in loss of its barrier function, a subsequent loss of membrane proteins and leakage of cellular material and an increase in the uptake of ordinarily membrane-impermeable material [107]. Further, HHPP has been shown to potentially induce some considerable changes in the bacterial cell such as inhibition of key enzymes, inhibition of protein synthesis, alterations in cell morphology, and may even possibly interrupt the genetic mechanisms of the microorganism responsible for survival and reproduction [105,106,107,108]. 

In spite of its considerable success in activation of *Salmonella* spp. in some other food items such as fruits, vegetables and meat, HHPP has shown low efficacy in other food products with high fat, protein and sugar content and low water activity such as peanut butter [109]. When creamy peanut butter was inoculated with *Salmonella* spp. at levels between 6 log–7 log CFU/g and subjected to HHPP of varying combination of pressures between 600 MPa and hold time of 18 min, only between 1.6 and 1.9 log CFU/g reduction in *Salmonella* spp. was achieved [76]. Again, in another experiment, 6 log to 7 log CFU/g *Salmonella* Typhimurium was inoculated in 3 samples of natural peanut butter brands and then HHPP treated at 600 MPa for 5 min at 45 °C, less than 1 log CFU/g reduction was obtained [103]. Higher efficacies (6 log CFU/g reduction) were only realised at much high water activity which would make peanut butter virtually unrecognisable [76,103]. In addition, the high lipid and protein content of peanut butter appeared to confer some protection to *Salmonella* spp. against HHPP inactivation [76,109]. While low water activity (a_w_) generally enhances the chances of survival of microbial cells against HHPP, microorganisms that are injured by HHPP are usually more sensitive to other stressors such as pH and solute concentration variations [107,108]. Thus, the solute (salt) concentration might be influential on the efficacy of HHPP when inactivation *Salmonella* spp. in peanut butter. Strictly speaking, and for all practical purposes, with respect to activation of *Salmonella* spp. in contaminated peanut butter, HHPP still has very limited success to date. However, better results could possibly be obtained in peanut sauces and syrups that are prepared at much higher water activity [110]. 

### 2.13. Use of Competitive and Antagonistic Bacterial to Inactivate Salmonella spp. in Contaminated Peanut Butter

In the future, probiotics may be used to inactivate *Salmonella* spp. in contaminated peanut butter. Both in vivo and in vitro, probiotics such as lactobacilli are known to produce H_2_O_2_, metabolites, and antimicrobial substances, including bacteriocins and other non-bacteriocin molecules which alters adhesiveness of bacterial pathogens such as *Salmonella* spp. in a way that enables them to inhibit the bacterial invasion in cultured epithelium cells [111]. Co-culture of Xynotyri cheese isolate *Lactobacillus plantarum* strain and *Salmonella* Typhimurium strain results in the substantial death of the pathogen [111]. While the actual effectiveness of inactivating *Salmonella* spp. in contaminated peanut butter still requires further study and validation, it is thus far further interesting to note that peanut butter has shown great potential as a carrier and vehicle for delivering of probiotics to improve gastrointestinal health [112,113]. When cocktails of commercial probiotic comprising of several strains of *Lactobacillus*, *Bifidobacterium*, *Streptococcus* and *Lactococcus* were inoculated in peanut butter at 10^7^ CFU/g and stored at 4 °C, the tested probiotic mixtures showed great survivability and viabilities over a 12-month storage period [112]. In another experiment probiotics fortified in peanut butter survived simulated gastrointestinal conditions and inhibited the growth of *Salmonella* spp. [113]. Given that peanut butter is a potentially stable carrier of probiotics, a study to evaluate the efficacy of the antagonistic relationship between *Salmonella* spp. and some probiotics within a peanut butter matrix might be worthwhile. 

### 2.14. Cleaning and Decontamination of Peanut Butter Plant Contaminated with Salmonella spp. 

Cleaning and decontamination of a peanut butter plant contaminated with *Salmonella* spp. is challenging. It has been observed that one of the most significant risk factors for *Salmonella* spp. contamination in low moisture food processing plants (such as peanut butter) is the presence of water, which allows for the proliferation of microorganisms, thereby increasing chances of product contamination [28]. If aqueous-based cleaning is employed, then extreme care must be taken to ensure that the plant is properly dried out of all the moisture as soon as is practically possible. Residual moisture might introduce another ecosystem of microbes that would have otherwise been absent given the natural low water activity of peanuts and peanut butter [29]. If residual moisture is allowed to persist in the plant, the potential for *Salmonella* spp. contamination of the peanut butter is greatly increased, thereby compromising peanut butter food safety. On the other hand, due to the oiliness of the peanut butter production plants, dry cleaning methods may even be problematic as well. In the absence of other validated non aqueous cleaning methods, for low moisture food plants, wet cleaning is sometimes generally restricted to situations only considered to be essential, in some cases only once a year [33]. There is also the general erroneous assumption that low moisture foods are at low risk of microbial contamination, as such some industrial plants and domestic peanut butter processing equipment may be used for longer periods without stopping for periodic cleaning and sanitizing [29]. In that case, the cross contamination of batches is highly likely. In the event of any associated foodborne outbreak which then might require the recall of all the associated products since the last date of cleaning of the facility, this can easily lead even to total bankruptcy of the concerned company [33]; such is the complexity of the problem *Salmonella* spp. contamination in a peanut butter processing plant. There is need therefore, to ensure that prevention, which is the first line of defence against *Salmonella* spp. contamination is always solid and by all means not breached at every stage of the production processes. 

A novel two-stage process of cleaning and disinfecting a *Salmonella* spp.-contaminated plant involving using hot oil followed by a non-aqueous based cleaning agent has been evaluated [29]. It was successfully demonstrated that a 2-step process involving hot oil flushing of the facility followed by 60% isopropanol or a mixture of isopropanol and quaternary ammonium compounds, is effective in cleaning peanut butter facilities contaminated with *Salmonella* spp. [29]. Furthermore, it was proved that hot oil flushing alone was not that effective in reduction in *Salmonella* spp. to acceptable levels in peanut butter processing plants. This is due to the fact that *Salmonella* spp. usually develops some heat resistance in low water activities environments. 

## 3. Food Safety Risk of Contamination of Peanut Butter by Aflatoxins

Mycotoxin contamination, specifically aflatoxins produced by the *Aspergillus* moulds, is another significant and important food safety risk that is associated with peanut butter consumption [114,115,116]. While in counties with robust food monitoring and strict food regulatory systems the risk of exposure might be controlled, countries are still struggling to manage [115,117,118,119]. If not properly monitored and controlled, the health impacts of aflatoxins contamination can be of quite substantial impacts [120,121]. Some notable examples include the cases of the ‘St Anthony’s fire’ pandemic of 943 AD, and the ‘Turkey X’ disease of 1978 [120]. Moreover, in 2004, an aflatoxin epidemic in Kenya claimed the lives of 125 people and further a total of 317 cases of acute liver failure were reported [122]. Williams, et al. [12] estimated that in developing countries, about 4.5 billion people are chronically exposed to largely uncontrolled amounts of aflatoxins. According to the World Health Organisation (WHO), the economic impact of aflatoxins on a global scale is such that it causes the destruction of about 25% of the world’s food crops annually [121]. For the greater part, human exposure to aflatoxins is through the consumption of nuts, grains, and their derived products, peanut butter included [121,122].

**Figure 2:**
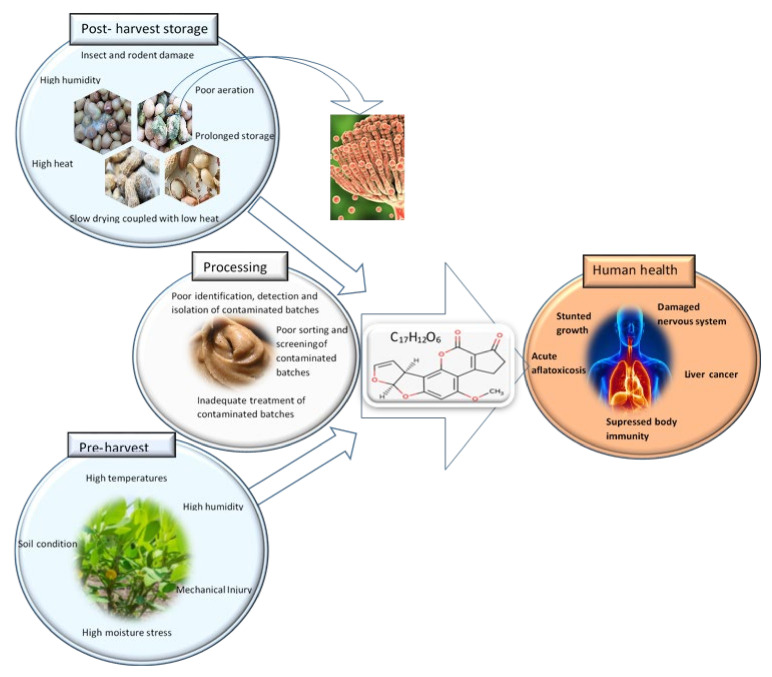
Causes and effects of aflatoxin B_1_ contamination in peanut butter. Poor pre-harvest and post-harvest storage conditions favour the development of aflatoxins in peanuts. Processing conditions can reduce aflatoxin contamination levels. Aflatoxins poisoning can result in acute and/or chronic health problems.

The health impacts of mycotoxins can be both acute or chronic, resulting in death or life-changing permanent damage to the central nervous system, suppression of the immune system, stranded growth, damages to the liver and hepatic system [119,123]. Aflatoxin B_1_ is the most prevalent and potent form of mycotoxins. It is most commonly found in corn and peanuts is also considered to be a class 1 carcinogen by the International Agency for Research on Cancer (IARC) [124]. Figure 2 shows the cause and effects of consumption of peanut butter contaminated with aflatoxin B_1_.

While the risk of exposure is almost equal for all age groups, the effects are more pronounced in infants and those with compromised immunity mostly those infected by HIV and Hepatitis [119,125]. Since it has been proven that aflatoxins are mutagenic in some bacteria, though not yet fully substantiated for humans, there is a likelihood that they can also contribute to birth defects in infants as well [119,125]. The fact that peanut butter is used extensively in developing country as cheap supplementary protein source for infants and those with compromised immunity further compounds this challenge. 

For peanuts and grains the Codex standards stipulates that the maximum level for aflatoxins contamination that is intended for consumption is 0.5 to 15 µg/kg [121]. With a large number of unmonitored and unregulated small scale peanut butter producers in developing countries, coupled with the conducive climatic conditions in the subtropics, the *Aspergillus* moulds growth in peanuts, and the risk of aflatoxins poisoning is more pronounced thus this recommended maximum standard is usually exceeded [114,117,124]. In one study reported in Kenya in 2010, of all the peanuts samples that were collected, 35% exceed the maximum allowable limit for that region which is set at 10 ppb [122]. Furthermore, in another study on the prevalence of peanut butter contamination in commercial peanut butter brands from Zambia, excessive-high levels of aflatoxin B_1_ were found [14]. Over the period of that study spanning from 2012 to 2014, at the very least, 53% of the 24 local and imported peanut butter brands from neighbouring sub-Saharan had concentrations of aflatoxins B_1_ greater than 20 μg/kg [14]. In some cases, concentrations as high as 10,740 μg/kg were observed. Furthermore, not even 1 of the 8 brands repeatedly tested across the 3-year period consistently averaged less than 20 μg/kg [14]. This shows that the problem of aflatoxin contamination in peanut butter is real and substantial, especially in developing counties. With the limited understanding and monitoring of aflatoxins, some health-related deaths in developing counties might be erroneously attributed to some other causes while in actual fact they can be traced back to peanut butter aflatoxin poisoning. 

### Control and Monitoring of Aflatoxin Contamination in Peanut Butter

According to the Food and Agriculture Organization of the United Nations (FAO) guidelines, the best way to avoid aflatoxins in peanut butter is to ensure that at pre-harvest and drying, contamination is avoided, and to put in place robust inspection and testing system for incoming batches of peanuts into the factory [117,120]. Conditions of high drought stress and insect damage creates cracks on the peanut shells which acts as entry points for the moulds [117,120]. Poor drying and storage of peanuts post-harvest can also be a significant contributing factor to aflatoxins development. Once contaminated to unacceptable levels, the subsequent removal of aflatoxins by processing is not guaranteed. Hence, best strategy lies in avoiding at all cost the processing of contaminated peanuts in the peanut butter production lines [120]. In the recent past, a biological, competitive exclusion strategy for reducing aflatoxin B_1_ contamination in peanuts using competitive, non-toxigenic strain of A. flavus in the soil of developing peanuts was tested [126]. It was observed that this strategy has the potential to reduce aflatoxin B1 by up to 85% [126]. It might be possible to try to adapt the methods that have been used to reduce aflatoxins in other foods such as ozone treatment and extrusion to reduce aflatoxin contamination in peanuts and peanut butter [127]. Gamma irradiation of peanut butter can also possibly retard the growth of mycotoxins. Notwithstanding the potential peanut butter oxidations problems that may emanate from this process, the free radicals (hydroxyl, and hydrogen peroxide) that are produced during gamma radiation have the potential to affect the DNA of the aflatoxin moulds and possibly result in their death [128,129]. Another strategy, though in its infancy involves consumption of a specialised clay (NovaSil) that is highly adsorptive of aflatoxins in the digestive tract. NovaSil has been shown to binds aflatoxin and prevents adsorption, metabolism, and subsequent aflatoxicosis in animals [130]. It has also been shown that lifetime exposure of NovaSil to rats was harmless. Successful human clinical trials could possibly go a long way in mitigating aflatoxicosis in those populations that are naturally exposed to high levels of aflatoxin [130].

To reduce the risk of consumer exposure to Aflatoxins, the European Commission in 2006 set up comprehensive guidelines to be observed by member states to ensure accuracy and standardisation of aflatoxins sampling [131]. Several methods for detecting and quantifying the levels of aflatoxin contaminations in food have been developed. Some of the most notable and prominent include high-performance liquid chromatography (HPLC), thin-layer chromatography (TLC), mass spectroscopy, enzyme-linked immune-sorbent assay (ELISA), and electrochemical immune sensor [132]. Notwithstanding their rapidity and simplicity, some methods such as ELISA and bright greenish-yellow fluorescence test are, however, recommended as screening tests only due to their limited degree of precision and accuracy in aflatoxins analysis [133,134]. Small scale peanut butter producers might not have adequate means and the infrastructure to test for aflatoxin contamination, as a result, WHO is currently working with some partners to develop low-cost, low technology, rapid and reliable methods for aflatoxins testing [121]. As a general guide, peanuts that appear to be mouldy, discoloured, or shrivelled should always be removed from the peanut butter production line [121]. It is very important for peanut butter producers to put in place efficient systems for monitoring and control of aflatoxins at the same time consumers should buy only known and reputable peanut butter brands with established efficient monitoring control standards.

## 4. Conclusions 

The peanut butter market is advancing and so is its popularity. It is now important to pay special attention to the food safety risks associated with the consumption of peanut butter. *Salmonella* spp., and aflatoxins contamination are the major food safety risks associated with the consumption of peanut butter in terms of prevalence and impacts on public health. The decontamination of peanut butter is, at the moment, difficult and the best way of risk mitigation is avoiding any contamination by all means possible. Regulators and manufactures must adopt and adhere to good manufacturing practices to minimise the risk of contamination. A significant amount of research on minimising and managing risk of peanut contamination is still required. 

## Figures and Tables

**Table 1:** Peanut butter standards for USA, Malaysia, and East African Community [66,67,68,69].

Specification	Country/Region		
	USA	Malaysia	East African Community
% peanuts (minimum)	90	85	90
% lipids (maximum)	55	55	55
% Salt (maximum)	1.6	2	2
% Moisture	-	3	2
Permitted additives			
% Stabiliser (maximum)	4	5	3
% Dextrose (maximum)	6	-	-

## Data Availability

The data presented in this study are available on request from the corresponding author.
